# RPL-4 and RPL-9 –Mediated Ribosome Purifications Facilitate the Efficient Analysis of Gene Expression in *Caenorhabditis elegans* Germ Cells

**DOI:** 10.1534/g3.120.401644

**Published:** 2020-09-03

**Authors:** Marco Nousch

**Affiliations:** Martin Luther University Halle-Wittenberg, Institute of Biology, Department of Developmental Genetics, 06120 Halle (Saale), Germany

**Keywords:** germ cell, translation, ribosomal protein, gene expression

## Abstract

In many organisms, tissue complexity and cellular diversity create a barrier that can hinder our understanding of gene expression programs. To address this problem, methods have been developed that allow for easy isolation of translated mRNAs from genetically defined cell populations. A prominent example is the Translating Ribosome Affinity Purification method also called TRAP. Here, ribosome associated mRNAs are isolated via purification of the ribosomal protein RPL10A/uL1, which is expressed under the control of a tissue specific promoter. Originally developed to investigate gene expression in mouse neurons, it has by now been adopted to many different organisms and tissues. Interestingly, TRAP has never been used successfully to analyze mRNA translation in germ cells. Employing a combination of genetic and biochemical approaches, I assessed several ribosomal proteins for their suitability for TRAP using the *Caenorhabditis elegans* germline as a target tissue. Surprisingly, I found that RPL10A/uL1 is not the ideal ribosomal component to perform such an analysis in germ cells. Instead other proteins such as RPL4/uL4 or RPL9/eL6 are much better suited for this task. Tagged variants of these proteins are well expressed in germ cells, integrated into translating ribosomes and do not influence germ cell functions. Furthermore, germ cell-specific mRNAs are much more efficiently co-purified with RPL4/uL4 and RPL9/uL6 compared to RPL10A/uL1. This study provides a solid basis upon which future germ cell TRAP experiments can be built, and it highlights the need for rigorous testing when adopting such methods to a new biological system.

The development of germ cells is driven by complex gene expression programs, which dictate the production of proteins to a large extent via post-transcriptional mechanisms. Two aspects make it challenging to perform a comprehensive analysis of germ cell gene expression programs in an organism. First, the tissue complexity of most multicellular organisms often limits the amounts of homogeneous biological materials that can be analyzed and methods that facilitate the enrichment for a specific cell population might introduce changes in the transcriptome (Richardson *et al*. 2015). Second, gene expression programs are usually analyzed by measuring mRNA abundances in a cell. However, especially developing female germ cells accumulate a large number of maternal mRNAs that are only translated into proteins after fertilization. Therefore, transcriptome measurements can only provide a limited understanding of the gene expression programs that drive germ cell development.

Two methods have been developed that aim at the characterization of the translatome in specific tissues named RiboTag and Translating Ribosome Affinity Purification (TRAP) (Sanz *et al.* 2009; Heiman *et al.* 2014). Both methods work with the same principle; ribosomes and their associated mRNAs are purified via a specifically tagged protein of the large subunit. The ribosomal protein (RPL) protein is expressed under the control of a tissue specific promoter. Both methods were originally designed to characterize gene expression in mouse neurons (Sanz *et al.* 2009; Heiman *et al.* 2014). The main difference between the two methods is the choice of the RPL protein. Whereas TRAP utilizes RPL10A/uL1, which was N-terminally fused to GFP, RiboTag uses RPL22/eL22, which was fused C-terminally to an HA-tag (Sanz *et al.* 2009; Heiman *et al.* 2014). Of the two methods only TRAP was adapted to other organisms, such as *C. elegans*, *D. melanogaster*, zebrafish and *xenopus* to investigate gene expression in a variety of tissues such as neurons, intestines and muscles (Thomas *et al.* 2012; Watson *et al.* 2012; Tryon *et al.* 2013; Gracida and Calarco 2017). However, neither RiboTag nor TRAP has been rigorously tested for their usability to analyze gene expression in germ cells.

Employing *C. elegans* as a model, the four ribosomal proteins RPL-1/uL1, RPL-4/uL4, RPL-9/uL6 and RPL-22/eL22 were tested for their TRAP-suitability in germ cells. The expression, developmental impact, integration into translating ribosomes and amounts of co-purified mRNA were analyzed from worm strains expressing the tagged ribosomal proteins. Surprisingly, this showed that the previously used RPL-1/uL1 and RPL-22/eL22 are out performed by RPL-4/uL4 and RPL-9/uL6, strongly implying that these two proteins are a much better choice for germ cell TRAP assays.

## Materials and methods

### Nematode strains and transgenesis

Worms were handled according to standard procedures and grown at 20°, if not otherwise stated (Brenner 1974). The N2 Bristol strain was used as a reference for wild type. The following strain was used in this study: LG *III: glp-1(q224)*. Transgenic strains EV850 (*efIs157**[Cbr-unc-119(+) + Pmex-5*::*rpl-9*::*FLAG*::*tbb-2 3′UTR] II*), EV927 (*efIs173**[Cbr-unc-119(+) + Pmex-5*::*rpl-1*::*FLAG*::*tbb-2 3′UTR] II*) and EV928 (*efIs174**[Cbr-unc-119(+) + Pmex-5*::*rpl-22*::*FLAG*::*tbb-2 3′UTR] II*) were generated using the Mos1-mediated single copy insertion (MosSCI) protocol (Frøkjær-Jensen *et al.* 2012). Injected constructs were assembled using the multisite Gateway cloning system (Thermo Fisher Scientific). To this end, the entire genes including introns for *rpl-1*, *rpl-9* and *rpl-22* were amplified from genomic DNA, fused with 3xFLAG-tag encoding sequences via overlap extension PCR, and inserted into the entry vector pDONR221. The assembled constructs were injected into the recipient strain EG6699 ttTi5605 II; unc-119(ed3) III; oxEx1578. EV484 (*efIs155[Cbr-unc-119(+) + Pmex-5*::*rpl-4*::*FLAG*::*tbb-2 3′UTR] II*) and was described previously (Nousch *et al.* 2019).

For the fertility analysis, L4 animals were singled and passaged to a new plate every 24hrs until the mother stopped laying embryos. Living larvae were counted to assess brood size.

### Western blotting

For Western blotting experiments samples were prepared from hand-picked worms, age 24h past mid L4, by boiling the collected material in Laemmli protein sample loading buffer prior to gel separation on 4–12% PAGE gradient gels (Eurogentec). Gel running and blotting was done following the manufacturer’s protocol. The composition of the blotting buffer was as followed: Tris base 15 mM and Glycine 192 mM. Western blots were visualized with an Odyssey Fc Imaging System (LI-COR) after incubation with IRDye secondary antibodies (LI-COR). All secondary antibodies were used at 1:10,000 dilutions in 5% Milk/PBS/0.05% Tween.

### Primary antibodies

Primary antibodies against the following proteins were used at the indicated dilutions: mouse anti-FLAG M2 (Sigma-Aldrich) 1:5000, anti-tubulin (T5168, Sigma) 1:5000 and RPS5 (sc-390935, Santa cruz) 1:100; and rabbit anti-PAB-1/2 (Nousch *et al.* 2014) 1:5000. All antibodies were diluted in 5% Milk/PBS/0.05% Tween.

### Extract preparation for immunoprecipitations and sucrose gradient centrifugation

Worms were synchronized at the L1 larval stage, grown on 10 cm MGM plates spotted with OP50 and harvested as young adults (24h past mid L4). For harvesting, worms were washed off the plates with M9, collected into 15 ml tubes and centrifuged for 2 min at 500xg. The worm pellet was washed twice with M9 and once with B70 buffer (HEPES pH 7.4 50 mM, KAc 70 mM, NaF 1 mM, β-glycerophosphate 20 mM, MgOAc 5 mM, Triton X-100 0.1% and glycerol 10%) using 5x the pellet volume. Finally, the pellet was resuspended in equal volume of B70, mixed carefully and droplets were made in liquid nitrogen. The resulting worm pearls were stored at -80°.

For extract preparation the frozen pearls were ground into a fine powder using a MR301 ball mill at 30 hertz (Retsch). The powder was resuspended in 300 µl of B70+Inhibitors (DTT 1 mM, PMSF 1 mM, Benzamidine 2 mM, Pepstatin A 1 µg/ml, Leupeptine 1 µg/ml, Pefabloc 0.1 µg/ml, RNAaseOUT 100 U/ml and Cycloheximide 100 µg/ml) and spun in a bench top centrifuge for 10 min at 10000xg and 4°. The clear supernatant was transferred into a clean tube and the protein concentration was measured. The extract was now ready to be used for sucrose gradient centrifugation or immunoprecipitations.

### Sucrose gradient centrifugation

Equal amounts of extracts were resolved through a 10 ml 17–50% sucrose gradient. The gradients were spun for 210 min at 35000 rpm and 4° in a SW40Ti rotor (Beckman Coulter). The fractionation was conducted bottom up while the absorbance profile at 260 nm was recorded. Proteins were concentrated by TCA precipitation and all pellets were dissolved in the same amount of Laemmli protein sample loading buffer.

### Immunoprecipitation of ribosomes

Cellular extracts were pre-cleared using Protein A agarose beads (Sigma-Aldrich) for 1h at 4°. Afterward, 200 µl of extract were incubated with 20 µl anti-FLAG M2 affinity agarose (Sigma-Aldrich) for 2h at 4° with gentle mixing. This was followed by washing the bead material three times with 300 fresh B70+Inhibitors. RNA was isolated from the matrix material as well as the input as described below.

### RNA isolation and qPCR

RNA was isolated from extracts or bead material using Trizol (Invitrogen). 200 ng of total RNA was reverse transcribed using random hexamer primers and ReverseAid Premium reverse transcriptase (Thermo Fisher Scientific), according to the manufacturer’s protocol. Quantitative PCR (qPCR) was conducted on an iQ5 (BioRad), using the ABsolute QPCR SYBR Green mix (Thermo Fisher Scientific) and gene-specific primers (sequences available upon request).

### Data availability

Strains and plasmids are available up on request. Supplemental material available at figshare: https://doi.org/10.25387/g3.12901601.

## Results

### Choice and expression of tagged ribosomal proteins

**Figure 1:**
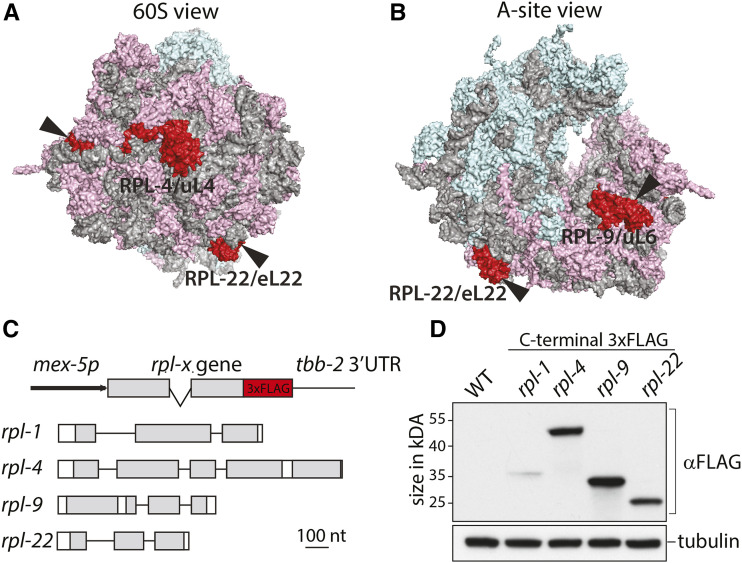
A differential expression is detected for tagged ribosomal proteins in germ cells. (A-B) Structure of the yeast 80S ribosome. RPL-4, RPL-9 and RPL-22 are shown in red. The arrow head indicates the position of the tag. Ribosomal RNA is shown in gray, proteins of the small subunit in light blue and proteins of the large subunit in pink. The original PyMOL file for the shown structure was generated by the Ban lab (https://bangroup.ethz.ch/research/nomenclature-of-ribosomal-proteins.html). (C) Top: Shown is the general structure of the expression constructs; Bottom: Genomic structure of *rpl-1*, *rpl-4*, *rpl-9* and *rpl-22*. Gray boxes indicate regions that define the different evolutionary conserved RPL protein regions. (D) Western blot analysis of RPL::FLAG expressing strains. Per lane 30 adult hermaphrodite were loaded.

Genes encoding homologs for all ribosomal proteins of the small and large subunit are present in the *C. elegans* genome (Table S1). The only exception is RPL41 for which no gene could be identified. Four proteins from the large subunit were chosen to be tested for their suitability in a potential germ cell TRAP assay, as mRNAs copurified with these proteins should be part of an 80S ribosome and therefore are most likely actively translated. I investigated RPL-1/uL1 and RPL-22/eL22, because they have been used in TRAP assays in the past (Heiman *et al.* 2008; Sanz *et al.* 2009). Furthermore, because of their size and position in the ribosome, I included RPL-4/uL4 and RPL-9/uL6. In general, RPL proteins are rather small and in *C. elegans* the median size of all RPL proteins is 16 kD. RPL-4/uL4 and RPL-9/uL6 can be considered large for RPL proteins, with ∼39 kD and ∼22 kDA respectively. These sizes should decrease the likelihood that a small tag interferes with the functions of the two proteins. Positional information about RPL-4/uL4, RPL-9/uL6 and RPL-22/eL22 within the assembled ribosome can be inferred from the crystal structure of the 80S complex from *Saccharomyces cerevisiae* (Ben-Shem *et al.* 2011). RPL-4/uL4 is located on the solvent side of the 60S subunit (Figure 1A), RPL-9/uL6 is close to the A-site (Figure 1B) and RPL-22/eL22 is close to the interface with the 40S subunit (Figure 1A and B). The position of RPL10A/uL1, the homolog of RPL-1, has been previously mapped close to the exit channel of the mRNA (Anger *et al.* 2013). In contrast to RPLs with strong integral binding positions within rRNA (*e.g.*, uL2, eL33 or eL37) RPL10A/uL1, RPL-4/uL4, RPL-9/uL6 and RPL-22/eL22 show more peripheral attachment to rRNA and should be accessible for immune-purifications.

Expression constructs were generated for FLAG-tagged RPL-1/uL1, RPL-4/uL4, RPL-9/uL6 and RPL-22/eL22 (Figure 1C). To this end, the genetic loci of the four *rpl*-genes were C-terminally fused to 3xFLAG and cloned into plasmids, which permit germ cell specific expression. The production of mRNAs was controlled by the well characterized *mex-5* promoter (*mex-5P*) and protein production was directed by the 3′UTR of tubulin (*tbb2*), which allows translation of an mRNA during all stages of germ cell development (Merritt *et al.* 2008). The entire expression cassettes were integrated into the *C. elegans* genome as single copies using the MosSCI method (Frøkjær-Jensen *et al.* 2012). Western blot analysis of homozygous adults that carry the expression cassettes revealed strong expression of tagged RPL-4/uL4 and RPL-9/uL6, moderate expression of tagged RPL-22/eL22 and low expression for tagged RPL-1/uL1 (Figure 1D). This shows that FLAG fusion proteins can be efficiently produced for RPL-4/uL4, RPL-9/uL6 and RPL-22/eL22 but not for RPL-1/uL1 in germ cells.

### Tagged ribosomal protein expression in germ cells has no influence on fertility

**Figure 2:**
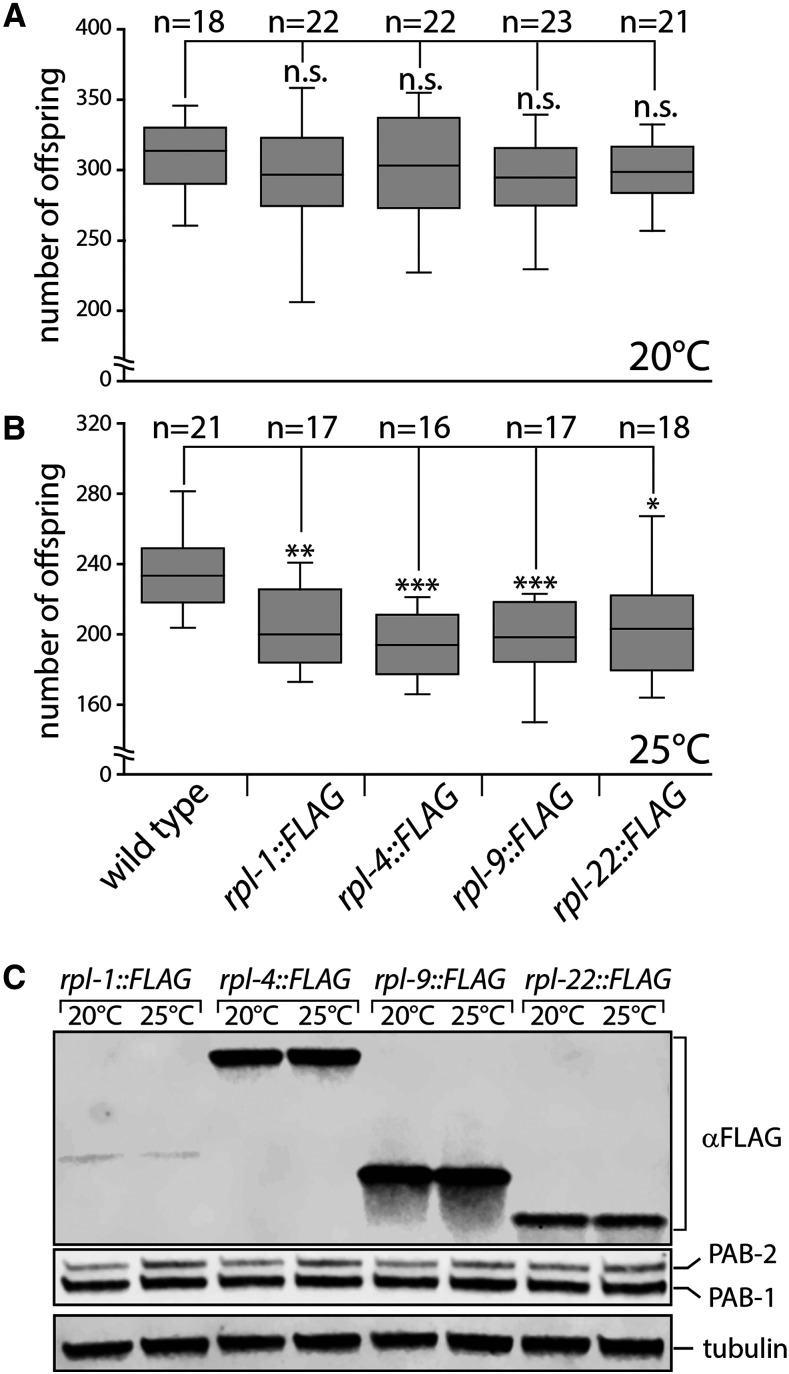
Expression of tagged RPLs in germ cells has no influence on fecundity. The fertility of parental hermaphrodites (n) was analyzed by counting hatched F1 larvae (progeny). (A) At 20°, no significant difference was detected in the number of median offspring generated by wild type or *rpl*::*flag* expressing worms. (B) At 25°, all *rpl*::*flag* expressing strains produce less progeny compared to wild type. (C) Western blot analysis of *rpl*::*flag* expressing strains grown at different temperatures. No obvious differences in expression levels are detected.

To test whether the expression of tagged RPL-1/uL1, RPL-4/uL4, RPL-9/uL6 or RPL-22/eL22 has an impact on germ cell development, I analyzed the brood sizes of homozygous transgenic animals grown at different temperatures. At 20°, a wildtype worm produces around 300 offspring (Figure 2A). No significant difference in brood size was detected for any of the animals expressing the tagged RPL proteins (Figure 2A). At 25°, a wildtype animal has a slightly reduced brood and produces around 250 offspring (Figure 2B). Interestingly, this number is significantly reduced to ∼200 progeny for all RPL::FLAG expressing strains (Figure 2B). To test whether this might be a consequence of increased expression of the tagged RPL proteins I investigated the RPL::FLAG protein levels by Western blotting. Surprisingly, no significant difference in RPL::FLAG expression could be detected between worms grown at 20° or 25° (Figure 2C). In general, no correlation seems to exist between RPL::FLAG expression levels and fertility, suggesting that the decrease in brood size at elevated temperature might be an inherent property of the MosSCI strain that was used in this work to generate the transgenic animals. This argues that the expression of tagged RPL-1/uL1, RPL-4/uL4, RPL-9/uL6 and RPL-22/eL22 has no negative impact on germ cell functions.

### Tagged RPL proteins are present in polysomes

**Figure 3:**
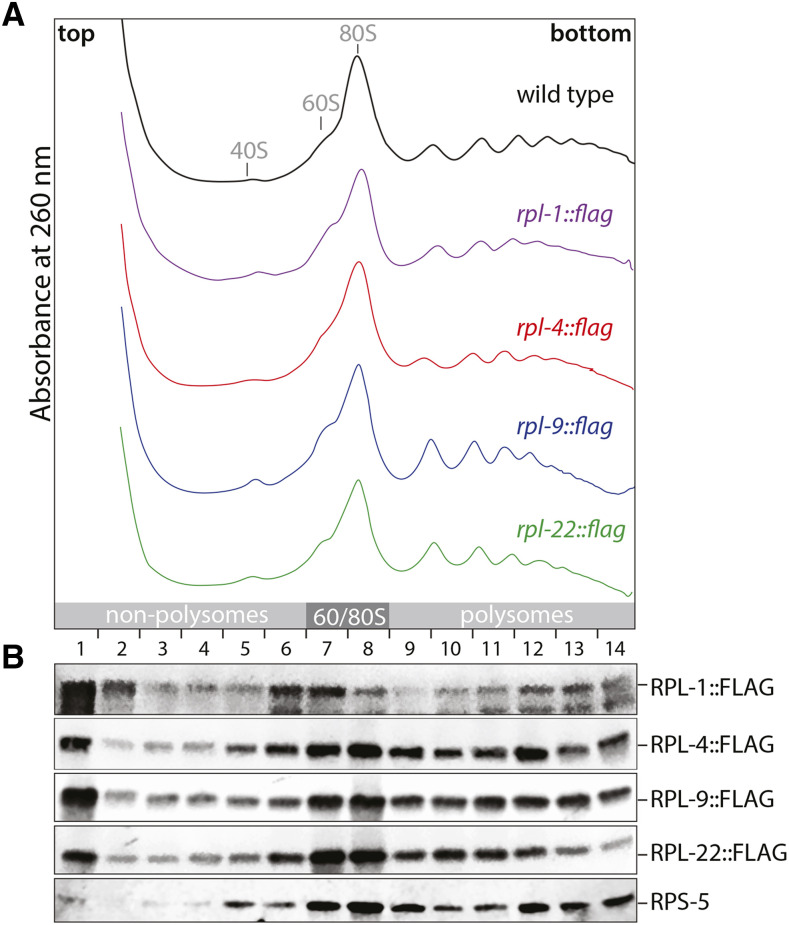
Differential integration of tagged ribosomal proteins into translating ribosomes. A polysome analysis was conducted comparing wild type to *rpl*::*flag* expressing animals. (A) Absorbance traces at 260 nm of polysomal gradients from wild type, *rpl-1*::*flag*, *rpl-4*::*flag*, *rpl-9*::*flag* and *rpl-22*::*flag* worms. Similar amounts of cellular proteins were loaded onto each gradient. The positions of the major ribosomal complexes are shown on the top. The numbers on the bottom indicate the fractions that were collected. (B) Western blot analysis of the gradient fractions. RPS-5 distribution was similar in all gradients. The image shown was generated from the *rpl-22*::*flag* gradient.

It is important that the tagged RPLs do not influence the efficiency of general translation. Hence, I conducted a polysome analysis investigating the distribution of active and non-active ribosomes (polysomes *vs.* non-polysomes). To this end, whole animal extracts from adult wild type or transgenic worms were separated on a 17–50% sucrose gradient and fractionated. During the fractionation procedure the absorbance at 260 nm was recorded in order to trace the migration pattern of ribosomes. In wild type, a prominent 80S ribosome peak was detected in the middle of the gradient dividing polysomal from non-polysomal regions (Figure 3A). Furthermore, only minor peaks corresponding to the 60S and 40S ribosomal subunits are present in this sample (Figure 3A). In the *rpl*::*flag* strains, the overall distribution of ribosomal complexes is highly similar to the one in wild type (Figure 3A). In adults, germ cells contribute a significant amount of biological material toward whole animal extracts. In order to judge how much RNA in lysate comes from germ cells, I measured total RNA amounts isolated from wild type and *glp-1**(**q224**)*, a temperature sensitive strain which virtually has no germ cells at 25° (Austin and Kimble 1987). The yield of isolated RNA from *glp-1**(**q224**)* adults grown at 25° was ∼50% lower compared to wild type (Fig. S1). This suggests that half of the total RNA in an adult originates from germ cells. Therefore, severe translational defects that occur in germ cells would be detectable in a polysome profile. Only small differences are detected among the different *rpl* samples. Heavy polysomes are slightly decreased and light polysomes are increased in *rpl-9*::*flag* and *rpl-22*::*flag* compared to *rpl-1*::*flag* and *rpl-4*::*flag* (Figure 3A). This minor shift in the distribution of translating ribosomes could indicate that FLAG-tagged RPL-9 and RPL-22 either slightly decrease translation initiation rates or increase translation elongation speeds. Nonetheless, the overall absence of strong polysome abnormalities argues that the expression of RP-1::FLAG, RPL-4::FLAG, RPL-9::FLAG or RPL-22::FLAG has no major effect on the global translation efficiency of ribosomes.

Next, I investigated whether the tagged RPLs are part of active translating ribosomes. Thus, the migration behavior of the fusion proteins in the gradients was analyzed by Western Blot. RPS-5 of the small ribosomal subunit served as a marker illustrating the gradient distribution of an endogenous ribosomal component. A small amount of RPS-5 is detected in fraction one, which is the top of the gradient (Figure 3B). This is most likely corresponding to free proteins, which are not associated with larger complexes and therefore do not enter the gradient. The majority of RPS-5 is present around the 80S peak and in polysomes (Figure 3B). The RPL::FLAG proteins accumulate stronger on top of the gradient compared to RPS-5 (Figure 3B). This might be explained by the fact that the fusion proteins have to compete with the endogenous versions of the RPLs for incorporation into the large ribosomal subunit. Regardless, all RPL::FLAG proteins in the gradient are detectable in 80S and polysomal fractions, mirroring to a large degree the distribution of RPS-5 (Figure 3B). This migration pattern suggests that all tagged RPLs can be incorporated into translating ribosomes. However, quantitative differences exist. RPL-1::FLAG is strongly present on the top and only weakly in heavy regions of the gradient. On the other hand, RPL-4::FLAG, RPL-9::FLAG and RPL-22::FLAG show robust co-sedimentation with polysomes, arguing that these three proteins are better integrated into functional ribosomes compared to RPL-1::FLAG (Figure 3B).

### Analyzing the ribosome association of germline mRNAs

**Figure 4:**
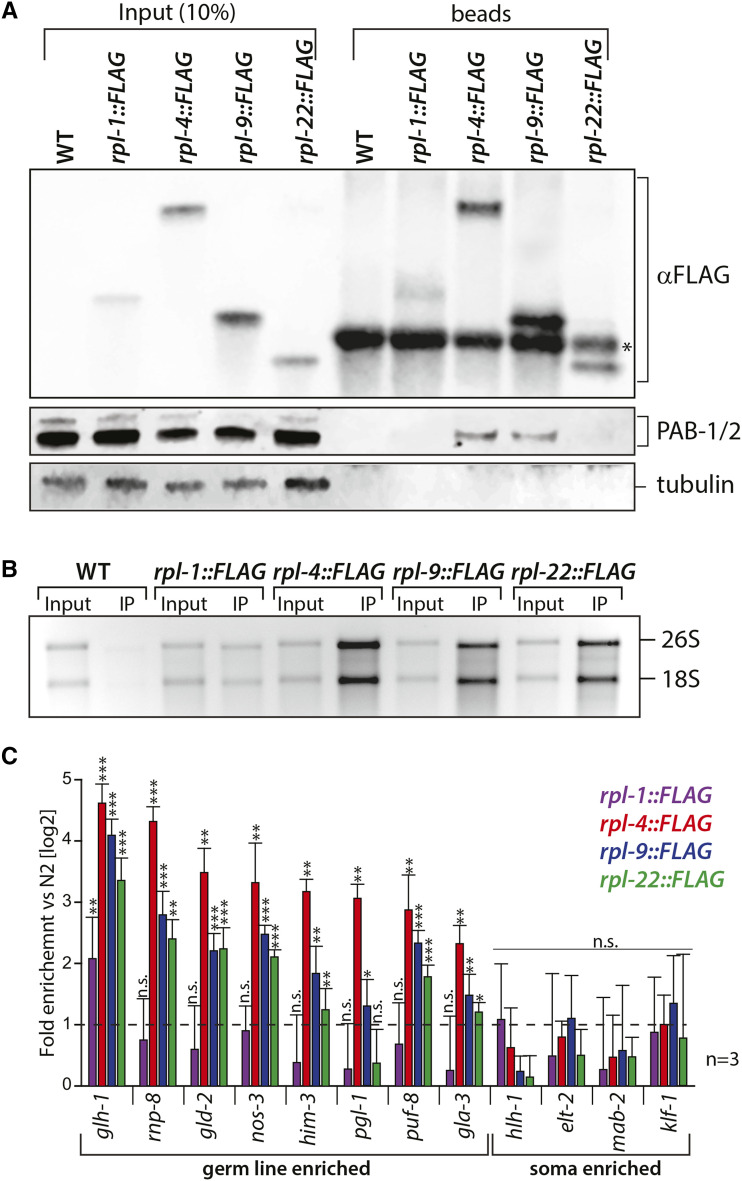
Translationally active mRNAs can be efficiently co-purified with RPL-4::FLAG and RPL-9::FLAG. (A) Western blot analysis of copurified RPL::FLAG proteins from adult worms. Equal amount of extract and anti-FLAG beads were used in the different immunopurifications. Input and bead material were analyzed for the indicated proteins. The asterisk in the FLAG blot markes an IgG band. (B) RNA was isolated from input and bead material and analyzed on a denaturing RNA gel stained with ethidium bromide for the presence of the 18S and 26S rRNA. Equal fractions of bead material were loaded for each immunoprecipitation. The signal in the input represents 1% of the amount of extract that was incubated with the bead material. (C) Analysis of mRNA enrichments in the different purifications using quantitative real time PCR.

Next, I addressed which of the different tagged RPL proteins can be utilized to analyze mRNA translation. To this end, the different tagged RPL proteins were purified from adult whole animal extracts using anti-FLAG beads. Extracts prepared from wildtype animals, which do not express a tagged protein, were also incubated with anti-FLAG beads and served as a negative control. For better quantitative comparison, equal amounts of extracts were incubated with the same bead volume for each sample. The investigation of the bead material by Western blotting shows that all tagged ribosomal proteins could be specifically enriched with the anti-FLAG beads (Figure 4A). The purification efficiency was low for RPL-1::FLAG, medium for RPL-22::FLAG and the highest for RPL-4::FLAG and RPL-9::FLAG (Figure 4A). To test whether translation-related proteins could be co-purified, the presence of cytoplasmic poly(A) binding protein PAB-1/2 was analyzed in the immune-purifications. PAB-1/2, a factor that binds to poly(A) tails and promotes translation, was detected in RPL-4::FLAG and RPL-9::FLAG purifications (Figure 4A) (Gu *et al.* 1995). On the other hand, tubulin, a protein not associated with translation, could not be detected in any purification (Figure 4A). In summary, all tagged RPLs can be purified using the FLAG tag. However, only RPL-4 and RPL-9 allow the efficient co-purification of a translational factor, suggesting that the two RPLs are part of active ribosomes.

Finally, it was tested whether components of the ribosome can be enriched with the tagged RPL proteins. To this end, RNA was isolated from the input and RPL::FLAG purified material and analyzed for the presence of ribosomal RNA. A signal for the 18S and 26S rRNA was barely visible in the control providing the baseline for a background enrichment of rRNA (Figure 4B). In all purifications rRNA was detected above background levels with the strongest signals in RPL-4::FLAG, RPL-9::FLAG and RPL-22::FLAG (Figure 4B). This suggests that in general ribosomes can be purified with all four tagged ribosomal proteins.

To test whether germline-specific mRNAs can be co-purified with the different RPL::FLAG proteins, RNA was isolated from purified material, converted into cDNA and the amounts of specific mRNAs were measured by quantitative real-time PCR. Eight mRNAs previously described as being germline-enriched and four mRNAs that are primarily expressed in the soma were quantified by measuring their enrichment with the different RPL::FLAG proteins relative to the control purification. None of the four soma-enriched mRNAs was significantly enriched with any RPL::FLAG purification (Figure 4C). In contrast to this, significant enrichments were detected for germline enriched mRNAs (Figure 4C). Again differences existed in co-purification efficiency, with RPL-1::FLAG showing enrichment for 1/8, RPL-4::FLAG for 8/8, RPL-9::FLAG for 8/8 and RPL-22::FLAG for 7/8 measured germline mRNAs (Figure 4C). This shows that a large complement of germ cell specific mRNAs can be efficiently isolated using tagged RPL-4, RPL-9 and RPL-22. The combined immunoprecipitation results strongly argue that tagging RPL-4 or RPL-9 provide the best handle to purify active ribosome/mRNA complexes from germ cells in *C. elegans*.

## Discussion

The goal of this work was to identify suitable ribosomal proteins that allow an efficient analysis of mRNA translation in *C. elegans* germ cells. Surprisingly, the overall data show that of the four tested RPLs the most commonly used in the literature, RPL-1/uL1, is not a good choice for this task. The RPL-1 fusion protein is not well expressed in germ cells, does not strongly associate with ribosomes and thus allows only inefficient copurification of germ cell mRNAs. The main difference between the RPL-1/uL1 variant in my work and studies in the past is the position and nature of the tag. Whereas in the presented work a C-terminal FLAG was used, the initial study in mice employed an N-terminally GFP-tagged RPL10A/uL1 for the purification procedure (Heiman *et al.* 2008). In the original work only GFP::RPL10A/uL1 was tested and nearly all studies that adopted the TRAP assay to other organism copied this design (Thomas *et al.* 2012; Watson *et al.* 2012; Tryon *et al.* 2013; Gracida and Calarco 2017). Only in zebrafish a double-tagged RPL10A with GFP on the N- and HA on the C-terminus was used to enrich hair cell-specific mRNAs (Matern *et al.* 2018). Unfortunately, the zebrafish study did not provide any evidence to which degree the double tagged protein is truly functional, and it is entirely possible that any modification at the C-terminus affects RPL-1/10A function. Therefore, using an N-terminal FLAG tag might improve the performance of an RPL-1 fusing protein for a TRAP assay in germ cells.

The TRAP method relies on the ectopic expression of modified ribosomal proteins in specific tissues and cells. For an easy adaptation of this assay to new biological systems the perfect TRAPable ribosomal protein should show little developmental expression variation. Interestingly, many ribosomal proteins have paralogs that can be expressed in a spatial and temporal restricted manner resulting in a tissue specific composition of ribosomes (Genuth and Barna 2018). Although, no functional paralogs have been described for uL1 and eL22 in *C. elegans*, these two proteins might contribute to the diversification of ribosomes in other organisms. In *Drosophila*, uL1 proteins are encoded by two genes, RpL10Aa and RpL10Ab (Wonglapsuwan *et al.* 2011). According to modENCODE data (http://www.modencode.org/), RpL10Ab seems to be the uL1 protein variant which is expressed in most tissues, whereas RpL10Aa expression is enriched in adult testis. For eL22, paralogs with tissue-specific functions have been described in flies and zebrafish (Zhang *et al.* 2017; Mageeney and Ware 2019). Contrary to this, rpl-4/uL4 and rpl-9/uL6 are each encoded by only one gene in worms, fly, fish and mice. This lack of protein variability argues that in all organisms these two RPLs should be easily suitable for ribosome purifications from most tissues.

Surprisingly, heterogeneity of the translational machinery might occur not only on the tissue level but also has been proposed to exist within cells. Absolute abundance measurements of 15 of the 80 core ribosomal proteins in polysomes from mouse embryonic stem cells showed that six proteins are substoichiometric (Shi *et al.* 2017). One of these six proteins is RPL10A/uL1, suggesting that not every actively translating ribosome contains this protein. Additionally, the same study found that RPL10A/uL1 purifications co-enrich for specific mRNAs (Shi *et al.* 2017). Interestingly, the vast majority of studies that characterize tissue-specific translatomes in organisms solely rely on data generated via RPL10A/uL1 purifications (Doyle *et al.* 2008; Heiman *et al.* 2008; Thomas *et al.* 2012; Zhou *et al.* 2013; Liu *et al.* 2014; Gracida *et al.* 2017; Matern *et al.* 2018; Rodrigues *et al.* 2020). This raises the question to which degree the TRAP data generated by these studies truly reflect the global translation programs occurring in the investigated cell types. Contrary to RPL-1/uL1, the proteins RPL-4/uL4, RPL-9/uL6 and RPL-22/eL22 are most likely constitutive and, thus, a part of every ribosome in a cell (Shi *et al.* 2017). Hence, mRNAs copurified via these proteins should provide an unbiased view of translation in cells.

A growing number of studies provide evidence that ribosomal proteins play an important part in the translation of specific mRNA (Kondrashov *et al.* 2011; Zhang *et al.* 2013; Shi *et al.* 2017). Hence, the view of the ribosome has shifted away from the rigid machine that is the same in every cell toward a more dynamic macromolecular complex with specialized compositions and functions (Genuth and Barna 2018). Consequently, methods originally developed to survey global mRNA translation in cells and tissues should be reevaluated, especially if adapted to a new biological system. The ideal ribosomal protein used for TRAP assays should be a stable component of the ribosome in many biological settings. The robust performance of RPL-4/uL4 and RPL-9/uL6 in *C. elegans* germ cells combined with the evolutionary conserved nature of uL4 and uL6 homologs as core components of the ribosome on the cellular as well as the tissue level, strongly argues that these proteins might be the ideal choice for future TRAP assays in many biological systems.
